# The importance of platelet counts in severe hypothermia

**DOI:** 10.1186/s12245-019-0234-y

**Published:** 2019-07-23

**Authors:** Ian Joseph Cohen

**Affiliations:** 1 Sackler Faculty of Medicine, Tel Aviv University, Ramat Aviv, Israel; 2 The Rina Zaizov Hematology-Oncology Division, Schneider Children’s Medical Center of Israel, Petach Tikva, Israel; 3 139 Shir Hashirim St, 44814 Elkanah, Israel

**Keywords:** Hypothermia thrombocytopenia, Rewarming deaths

To the editor,

I would like to congratulate Bunya et al. for their case report of a successful recovery of a patient after severe hypothermia published recently in the journal [1]. It is rewarding to see that their dedication and refusal to assume that the patient was dead resulted in successful recovery. I would like to comment on what may seem to be a minor detail in the report but would seem to me to be very significant. The authors report that the platelet count on admission was 14.7 × 104/μl, i.e., 147,000/mm^3^. This is a very unusual finding in severe hypothermia since thrombocytopenia is to be expected in this condition (especially in a patient with a core temperature as low as 22 °C) and is the basis of the bleeding complications that may be responsible for “rewarming deaths.” We have described this in infants who have become hypothermic and have shown that the thrombocytopenia becomes much more marked on rewarming due to priming of the platelets by the cold [2, 3]. We have shown (in blood from adults) that below 30 °C the second phase of platelet aggregation and release of dense granules is blocked but that rewarming causes massive activation of platelets.

Aspirin has been shown to prevent these rewarming changes [4], and other compounds have been shown to affect platelet function in a similar way. A possible explanation for the lack of thrombocytopenia in this case is the “premedication” the patient took with sleeping tablets [5] and alcohol [6] both of which may well have prevented both the thrombocytopenia and also the deepening of the platelet count on rewarming and thus avoided this potentially fatal complication during recovery. It is important that the treating physicians of other patients without such “premedication” are aware of the importance of following the platelet counts especially as the patients are being rewarmed, since giving platelets at that stage has been shown to prevent any bleeding complications [2] and a lack of awareness of the importance of thrombocytopenia may result in unexplained mortality.

## Data Availability

Data sharing is not applicable to this article as no datasets were generated or analyzed during the current study.
